# Changes in Gut Microbiome Following Acupuncture and Moxibustion in Patients With Parkinson Disease: Protocol for a Single-Group, Prospective, Observational Study

**DOI:** 10.2196/76551

**Published:** 2025-10-29

**Authors:** Han-Gyul Lee, Seungwon Kwon, Mijung Yeom, Sun-Young Bae, Hi-Joon Park

**Affiliations:** 1 Department of Cardiology and Neurology Kyung Hee University College of Korean Medicine Kyung Hee University Medical Center Seoul Republic of Korea; 2 Acupuncture and Meridian Science Research Center Kyung Hee University College of Korean Medicine Seoul Republic of Korea; 3 Department of Medical Science of Meridian Graduate School Kyung Hee University Seoul Republic of Korea

**Keywords:** neurodegenerative disorders, gut-brain axis, microbial dysbiosis, motor and nonmotor symptoms, complementary medicine

## Abstract

**Background:**

Parkinson disease (PD), a prevalent neurodegenerative disorder characterized by motor and nonmotor symptoms, is becoming increasingly prevalent worldwide. Conventional treatment for PD involves dopamine therapy, including levodopa; however, this treatment is ineffective for nonmotor symptoms and may cause adverse effects. The gut-brain axis has been hypothesized to promote PD, and regulation of gut microbiome, which modulates the gut-brain axis, is emerging as a treatment target. Acupuncture and moxibustion exert therapeutic effects on PD and modulate the gut microbial composition.

**Objective:**

We present a protocol for analyzing the effects of acupuncture and moxibustion on gut microbiome and exploring its association with symptoms in patients with PD.

**Methods:**

This single-group, prospective, observational study will recruit 60 patients with idiopathic PD and 20 healthy participants. Baseline gut microbiome patterns and motor and nonmotor symptoms of both groups will be compared. Patients with PD will be treated with acupuncture, moxibustion, and intradermal acupuncture twice a week for 12 weeks (24 sessions total). Motor and nonmotor symptoms and gut microbiome changes in patients with PD will be compared before starting treatment (day 0), during treatment (6 weeks), at the end of treatment (12 weeks), and 2 months after the end of treatment (20 weeks). The correlation between motor and nonmotor symptoms of PD changed by acupuncture and moxibustion treatment and changes in gut microbiome will be analyzed. Healthy participants will be assessed for motor and nonmotor symptoms of PD and gut microbiome after screening.

**Results:**

This study was supported by the National Research Foundation of Korea, funded by the Ministry of Science and ICT (Information and Communication Technology), Republic of Korea, and recruitment for the study started on October 21, 2021. As of February 19, 2025, recruitment and observation ended, and data analysis is being conducted.

**Conclusions:**

This is the first clinical study to assess the effects of acupuncture and moxibustion on gut microbiome and explore its association with symptoms in patients with PD. The results will provide clinical evidence to explain the microbiome-gut-brain axis mechanism of acupuncture and moxibustion for PD and suggest the possibility of acupuncture as an alternative therapy for PD.

**Trial Registration:**

Clinical Research Information Service KCT0006669; https://tinyurl.com/42jsxs5a

**International Registered Report Identifier (IRRID):**

DERR1-10.2196/76551

## Introduction

Parkinson disease (PD) is the second most common neurodegenerative disorder [1], and its incidence has increased drastically worldwide over the past few decades, placing it as one of the most rapidly expanding neurological disorders worldwide [2,3]. The rapid growth of PD is influenced by global aging, as the prevalence of PD increases with age [4]. Therefore, the development of treatment strategies for patients with PD is expected to become increasingly important.

PD is characterized by motor symptoms such as tremor, rigidity, bradykinesia, and postural instability due to altered aggregation of α-synuclein in the brain structures termed Lewy bodies, as well as by early prominent death of dopaminergic neurons in the substantia nigra [5]. However, PD is not only characterized by motor symptoms but also by nonmotor symptoms such as constipation, depression, anxiety, anosmia, pain, and sleep behavior disorder, which have been found to occur in the prodromal phase before the onset of motor symptoms, contributing to the decreased quality of life in affected patients [6]. Based on this knowledge, a study has recently suggested that PD should be approached as a systemic disease, rather than simply as a movement disorder caused by brain dysfunction [7].

Levodopa symptomatic therapy based on conventional brain pathology is the current mainstay of PD treatment [8]. However, nonmotor symptoms are known to be unresponsive to levodopa [9], and excessive and prolonged therapy is associated with a risk of adverse events, such as dopamine dysregulation syndrome [10] and levodopa-induced dyskinesia [11]. Therefore, current treatments that target the brain alone are unlikely to be effective in treating PD, indicating the need for alternative treatments.

The gut-brain axis theory posits that the gut and brain communicate bidirectionally with each other via the blood and vagus/sympathetic nerves to regulate various human physiological and pathological functions, and the gut microbiome is gaining attention as a potential modulator of the gut-brain axis [12]. As the highly complex relationship between the gut and brain in PD has been recognized, the role of gut microbiome as a modulator of PD has also been highlighted [13].

In Traditional East Asian Medicine (TEAM), both acupuncture and moxibustion have a long history of clinical use in the treatment of PD. Recently, there has been a growing demand for complementary and alternative medicine (CAM) for PD worldwide, to compensate for the limitations of conventional PD treatments, with acupuncture playing a large role [14]. In a previous review, acupuncture and moxibustion were reported to be superior to dopamine monotherapy in improving motor and nonmotor symptoms and managing long-term symptoms in PD [15]. In another study [16], the addition of acupuncture to dopamine replacement therapy was associated with significantly superior Unified Parkinson's Disease Rating Scale (UPDRS) I-IV score improvements compared with those noted with dopamine replacement therapy alone.

Recent experimental studies have shown that acupuncture modulates the gut microbial composition [17,18]. Based on this evidence, a study using an in vivo PD model reported that GB34 and ST36 acupuncture enhanced motor symptoms and dopamine neuron protection by modulating gut microbial dysbiosis and inhibiting neuroinflammation [19]. This suggests that the effects of acupuncture may also be mediated by modulating the gut-brain axis through gut microbiome regulation. However, all studies conducted in this field to date have been experimental, highlighting the need to explore the effects of acupuncture and moxibustion on gut microbiome and explore its association with clinical symptom improvement in patients with PD.

Herein, we present a protocol for a study analyzing the effects of acupuncture and moxibustion on the gut microbiome and exploring its association with symptoms in patients with PD. This study will provide new clinical evidence on the mechanisms of PD treatment with acupuncture and moxibustion, thereby increasing the level of evidence for acupuncture and moxibustion as alternative treatment methods for PD.

## Methods

### Study Design

**Figure 1:**
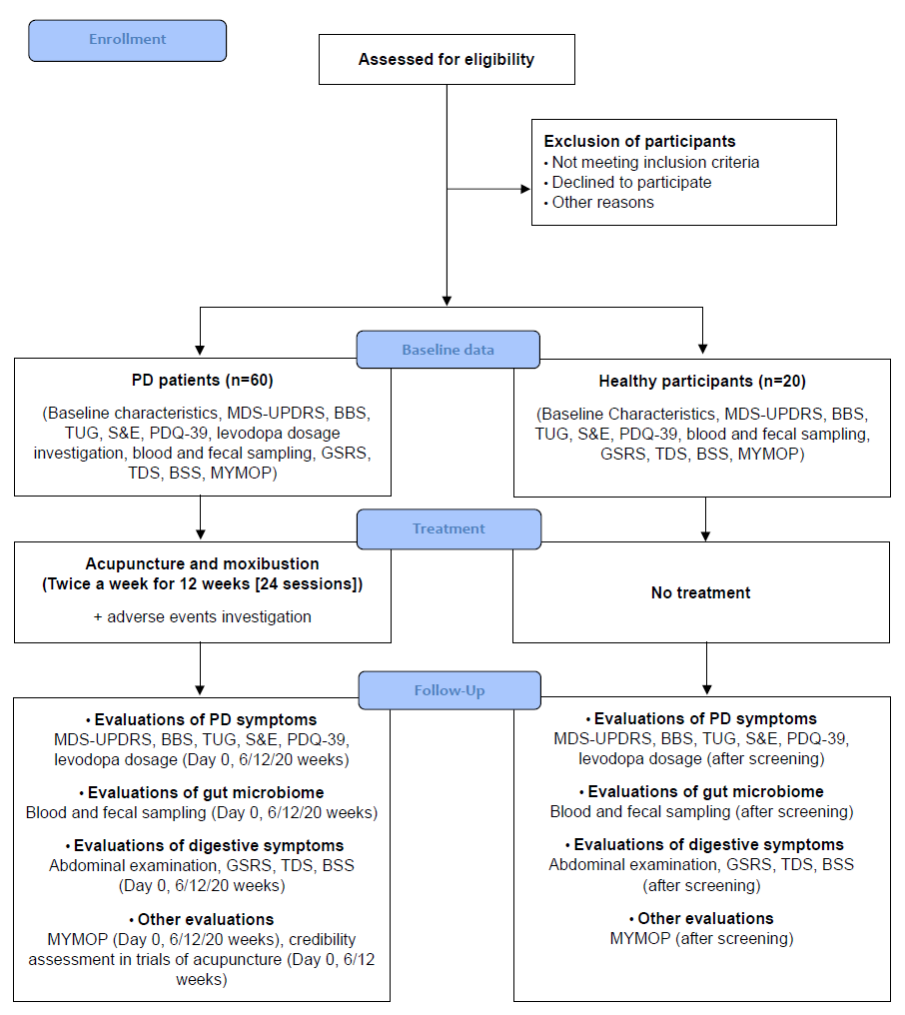
Flowchart of the study process. BBS: Berg Balance Scale; BSS: Bristol Stool Scale; GSRS: Gastrointestinal Symptom Rating Scale; MDS-UPDRS: Movement Disorder Society–Sponsored Revision of the Unified Parkinson's Disease Rating Scale; MYMOP: Measure Yourself Medical Outcome Profile; PD: Parkinson disease; PDQ-39: 39-Item Parkinson's Disease Questionnaire; S&E: Schwab and England Activities of Daily Living Scale; TDS: Total Dyspepsia Symptom Scale; TUG: Timed Up and Go Test.

This protocol describes a single-group, prospective, observational study aiming to analyze differences in gut microbiome between patients with idiopathic PD and healthy participants, to observe changes in gut microbiome after 12 weeks of acupuncture and moxibustion in patients with PD, and to correlate these changes with changes in motor and nonmotor symptoms of PD that were altered by acupuncture and moxibustion treatment. The gut microbiome patterns at baseline in healthy participants and screened patients with PD will be analyzed to determine whether the gut microbiome of patients with PD is different from that of healthy participants. Patients with PD will be treated with acupuncture, moxibustion, and intradermal acupuncture twice a week for 12 weeks, yielding a total of 24 sessions. The motor and nonmotor symptoms of PD and gut microbiome changes in response to acupuncture and moxibustion will be compared before starting treatment (day 0), during treatment (6 weeks), at the end of treatment (12 weeks), and 2 months after the end of treatment (20 weeks). The correlation between motor and nonmotor symptoms of PD changed by acupuncture and moxibustion treatment and changes in gut microbiome will be analyzed. The enrollment, interventions, and assessments of patients with PD are summarized in Table S1 in Multimedia Appendix 1. A schematic of the study process is shown in Figure 1. Healthy participants will be assessed for motor and nonmotor symptoms of PD and gut microbiome after screening. The enrollment and assessment of healthy participants are summarized in Table 1. Kyung Hee University Korean Medicine Hospital, Seoul, Republic of Korea, will serve as the study site. The study protocol was registered with the Clinical Research Information Service (KCT0006669) on October 15, 2021. This protocol was reported in accordance with the SPIRIT (Standard Protocol Items: Recommendations for Interventional Trials) guidelines (Multimedia Appendix 2).

**Table 1:** Schedule for the enrollment, interventions, and assessments of healthy participants.

Visit	0	1
Day	–14 to 0	0
Week	0	1
Visit window	—	±7
Informed consent	✓	
Demographic information	✓	
Medical history	✓	
Medications investigation	✓	
Vital sign	✓	
Hoehn and Yahr stage	✓	
Evaluating inclusion and exclusion criteria	✓	
MDS-UPDRS^a^		✓
BBS^b^		✓
TUG^c^		✓
S&E^d^		✓
PDQ-39^e^		✓
Blood sampling^f^		✓
Fecal sampling^g^		✓
Abdominal examination		✓
GSRS^h^		✓
TDS^i^		✓
BSS^j^		✓
MYMOP^k^		✓
Korean medicine doctor interview		✓

^a^MDS-UPDRS: Movement Disorder Society–Sponsored Revision of the Unified Parkinson's Disease Rating Scale.

^b^BBS: Berg Balance Scale.

^c^TUG: Timed Up and Go Test.

^d^S&E: Schwab and England Activities of Daily Living Scale.

^e^PDQ-39: 39-Item Parkinson's Disease Questionnaire.

^f^Analyzing gut microbiome–related metabolites.

^g^Analyzing gut microbiome.

^h^GSRS: Gastrointestinal Symptom Rating Scale.

^i^TDS: Total Dyspepsia Symptom Scale.

^j^BSS: Bristol Stool Scale.

^k^MYMOP: Measure Yourself Medical Outcome Profile.

### Eligibility

Inclusion and exclusion criteria for patients with PD are summarized in Textbox 1.

Since patients with early-stage PD typically show a good response to anti-Parkinson agents such as levodopa [20], it is challenging to isolate and evaluate the additional effects of acupuncture and moxibustion in this population. Therefore, this study included patients who have been taking anti-Parkinson agents for more than 5 years or who were at Hoehn and Yahr stage 2-3, representing the midstage or beyond. Inclusion and exclusion criteria for healthy participants are summarized in Textbox 2.

Dropout criteria are summarized in Textbox 3.

**Inclusion and exclusion criteria for patients with Parkinson disease (PD).**

**Inclusion criteria**

- Men and women aged 50-85 years
- Patients with idiopathic PD who have been taking anti-Parkinson agents (excluding anticholinergic drugs) for >5 years
- Hoehn and Yahr stage 2-3
- Patients who have no cause of cognitive or motor impairment other than PD
- Patients who have voluntarily agreed to participate
- Patients who have not participated in other clinical trials within the last 1 month

**Exclusion criteria**

- Patients diagnosed with secondary Parkinsonism or atypical Parkinsonian syndrome
- Consumption of oral antibiotics that may affect results within the past month
- Loss of consciousness for more than an hour due to head trauma
- Patients with severe vision or hearing problem
- Consumption of probiotics that may affect results within the past month
- Consumption of medication (such as analgesics, anti-inflammatory drugs, or antibiotics) that may affect the gastrointestinal system within a week from the time of fecal sampling
- History of severe heart disease (eg, myocardial infarction or heart failure)
- Patients with a medical history of neurosurgical procedure (eg, deep brain stimulation)
- Patients who have been diagnosed with cancer or treated for cancer
- Patients with glaucoma
- Chronic alcohol consumption or drug abuse
- Women who are pregnant or breastfeeding
- Patients with diseases such as coagulation disorders that cannot be treated with acupuncture
- Patients with clinically significant psychiatric symptoms or medical diseases
- Participants who are judged by the investigator to have nonconformity for participation in the trial for any reason

**Inclusion and exclusion criteria for healthy participants.**

**Inclusion criteria**

- Men and women aged 50-85 years
- Participants who have no problems with cognitive function and who have voluntarily agreed to participate in the study through written consent
- Participants who selected to match the “Patients with PD” group with age, sex, and other health conditions, excluding the diagnosis of Parkinson disease (PD)

**Exclusion criteria**

- Participants diagnosed with PD
- Participants who have taken oral antibiotics within the past 1 month, as it was assumed that this medication could affect the results of this study
- Participants with a history of loss of consciousness for more than 1 hour due to brain trauma
- Participants with vision or hearing problems to the extent that the examination cannot proceed smoothly
- Participants who have consumed health functional food such as Lactobacillus within the last 1 month, as it is assumed that this health functional food could affect the results of this study
- Participants who took medications that could affect the gastrointestinal tract, such as pain relievers, anti-inflammatory drugs, and antibiotics, within 1 week of stool collection
- Participants with a history of serious heart disease such as myocardial infarction or heart failure
- Participants with a history of malignant tumor
- Participants with a history of glaucoma
- Participants with a history of chronic alcohol or substance abuse
- Participants with clinically significant psychiatric symptoms or medical conditions
- Any participant that the investigator judged to be physically or mentally inappropriate to participate in the clinical trial based on other laboratory findings

**Dropout criteria.**

**Dropout criteria**

- Participants who do not comply with the study physician's instructions
- Participants who have taken other medications that may affect the results of the study without the consent of the study physician
- Patients who have developed other secondary infections and disease conditions that may affect Parkinson disease symptoms
- Participants who have withdrawn consent or requested discontinuation of treatment

### Recruitment

For the study, 20 healthy participants and 60 patients with PD will be recruited through walk-ins or hospital and school bulletin boards, public transportation billboards, newsletters, and other online advertisements. Participants who wish to participate will receive an explanation of the consent form, provide written consent of their own free will, and participate in the study if the researcher determines that they are suitable through a screening process. The screening items are as follows: demographic information, medical history, PD diagnosis, treatment history, medications, vital signs, Hoehn and Yahr stage assessment, and inclusion and exclusion criteria evaluation (Table S1 in Multimedia Appendix 1 and Table 1 on code rather than the participant's name.

### Interventions

All patients with PD will receive acupuncture, moxibustion, and intradermal acupuncture treatments twice a week for 12 weeks, for a total of 24 sessions.

#### Acupuncture Treatment

Acupuncture treatment for patients with PD will be performed by a Korean medicine doctor specializing in internal medicine of Korean medicine or by a Korean medicine doctor with at least 2 years of clinical experience in the field under the supervision of a specialist, using disposable sterile acupuncture (Dongbang Acupuncture, Co, Ltd) with a thickness of 0.25 mm and a length of 30 or 40 mm. The acupuncture points will be 12 basic acupuncture points and 36 additional acupuncture points selected according to the relevant nonmotor symptoms (Table 2). A total of 25 points or more on both sides will be chosen. After inserting the needle vertically approximately 5-30 mm deep into each point and inducing qi with twirling for 10-15 seconds, the needle will be retained for 15 minutes and removed. All acupunctur*e* treatments will be performed in compliance with the STRICTA (Standards for Reporting Interventions in Clinical Trials of Acupuncture) guidelines [21].

**Table 2:** Acupuncture points planned for use in patients with Parkinson disease.

Type	Acupuncture points
Basic acupuncture points	Bai hui (GV20), bilateral Fengchi (GB20) Upper limb: Hegu (LI4), Zhongzhu (TE3), Quchi (LI11), Shousanli (LI10), Waiguan (TE5) Lower limb: Yanglingquan (GB34), Zusanli (ST36), Xuanzhong (GB39), Taichong (LR3), Zulinqi (GB41) Auricular acupuncture: Shenmen
Additional acupuncture points selected according to nonmotor symptoms	Constipation: Bilateral Tianshu (ST25), Yanggu (SI5), Yangxi (LI5) Frequent urination, dysuria, sexual dysfunction: Jingqu (LU8), Taibai (SP3), Taixi (KI3), Fuliu (KI7) Sialorrhea: Shaofu (HT8), Dadu (SP2), Dadun (LR1), Yinbai (SP1) Dysgeusia, dysosmia: Shangxing (GV23), bilateral Yingxiang (LI20) Pain: Ouch point Sleep disorder: Neiguan (PC6), Shenmen (HT7), Zhaohai (KI6), Shenmai (BL62) Anxiety, depression: Jingqu (LU8), Zhongfeng (LR4), Yingu (KI10), Ququan (LR8) Fatigue: Qihai (CV6), Guanyuan (CV4), Zhongji (CV3) (moxibustion) Cognitive impairment: Shishencong Dysphagia: Chengjiang (CV24), Lianquan (CV23), Danzhong (CV17) Dyspepsia: Zhongwan (CV12), Gongsun (SP4) Dizziness: Neiguan (PC6), Shenmun (HT7), Lieque (LU7) Fenglong (ST40), Taibai (SP3)

#### Electric Moxibustion Treatment

Using electric moxibustion (Onttum, TechnoScience Co, Ltd), heating stimulation will be applied to the bilateral Tianshu (ST25), Zhongwan (CV12), Qihai (CV6), Guanyuan (CV4) at 40 °C mode for the duration of the acupuncture treatment (15 minutes).

#### Intradermal Acupuncture Treatment

After acupuncture and moxibustion treatments are completed, intradermal acupuncture (Press Needle, Dongbang Medical Co, Ltd) will be attached to the bilateral Yanglingquan (GB34), Zusanli (ST36), and auricular Shenmen. Patients will be instructed to frequently apply acupressure to the attachment area.

### Outcome Measurements

#### Evaluation Overview

The efficacy of acupuncture and moxibustion in managing the motor and nonmotor symptoms of PD will be evaluated using the Movement Disorder Society–Sponsored Revision of the Unified Parkinson's Disease Rating Scale (MDS-UPDRS), Berg Balance Scale (BBS), Timed Up and Go Test (TUG), Schwab and England Activities of Daily Living Scale (S&E), 39-Item Parkinson's Disease Questionnaire (PDQ-39), and levodopa dosage investigation. To assess the association between acupuncture treatment-induced changes in symptoms and gut microbiome changes in PD, we will investigate changes in gut microbiome–related metabolites through blood sampling and changes in gut microbiome through fecal sampling. Participants’ digestive symptoms will be assessed by abdominal examination, Gastrointestinal Symptom Rating Scale (GSRS), Total Dyspepsia Symptom Scale (TDS), and Bristol Stool Scale (BSS). Subjective perception of health will be assessed using the Measure Yourself Medical Outcome Profile (MYMOP). Credibility assessment in trials of acupuncture will be conducted to evaluate the impact of psychological factors on patients with PD.

#### Evaluation of Motor and Nonmotor Symptoms of PD

The MDS-UPDRS, the primary outcome of this study, was developed to complement the UPDRS as a tool to assess motor and nonmotor symptoms in patients with PD [22], and the Korean version has been validated [23]. The MDS-UPDRS comprises the following four parts with a total of 65 items: (1) nonmotor experiences of daily living, (2) motor experiences of daily living, (3) motor examination, and (4) motor complications. Of these, 48 items are scored from 0 to 4, with 0 (robust), 1 (slight), 2 (mild), 3 (moderate), and 4 (severe), and the 17 remaining items are scored as yes or no. Scores are calculated by summing the scores for each item, with higher scores indicating more severe symptoms of PD. The BBS is used to assess balance [24] and is also useful in evaluating equilibrium in patients with PD [25], and the Korean version of this tool has been validated [26]. This tool comprises 14 items that are scored from 0 (worst) to 4 (best), and the total score is evaluated. The TUG is used for assessing gait in older adults [27] and is used to evaluate walking ability in patients with PD [28]. This test measures the time taken to get up from a chair, walk around a 3-meter return point, and return to the chair in the initial position. The S&E is a component of the UPDRS that evaluates functional independence and is widely used as a standard Parkinson assessment tool [29]. The patient’s function is evaluated using an 11-point scale with 10% increments, from 100% (completely independent) to 0% (vegetative functions are not functioning or bedridden). The PDQ-39 is used for assessing health-related quality of life (HrQoL) in patients with PD [30], and the Korean version has been validated [31]. The PDQ-39 comprises 8 dimensions with 39 items. Each item is scored from 0 (never) to 4 (always), with a higher total score indicating poorer HrQoL due to PD. Levodopa dosage investigation includes assessment of the daily dose and number of levodopa doses administered to patients with PD. Changes in type and dose of levodopa from pretreatment will be investigated first as “yes or no” and then dichotomized into “decrease or increase” for participants with no change for comparison between groups. Evaluation will be conducted at day 0, 6 weeks, 12 weeks, and 20 weeks in participants with PD and after screening in healthy participants.

#### Evaluation of Gut Microbiome–Related Metabolites and Gut Microbiome Change

Blood samples will be used to assess changes in gut microbiome–related metabolites. Blood will be collected from participants after an 8-hour fast before blood collection, and changes in metabolites will be determined using liquid chromatography-mass spectrometry and gas chromatography-mass spectrometry analyses. Western blotting will be applied to confirm changes in strain-derived factors in the blood of patients with PD. Participants’ fecal samples will be used to analyze the composition of the gut microbiome using 16S rRNA sequencing and fecal microbiome transplantation to assess the correlation between gut microbiome and response to acupuncture treatment. A small amount of feces will be collected using 4N6 FLOQSwabs (Copan Diagnostics Inc). For participants with PD, 2-3 g of feces will be collected using a Stool Collection Tubes With DNA Stabilizer (Invitek Diagnostics) for fecal microbiome transplantation. A FastDNA SPIN Kit for Feces (MP Biomedicals) will be used to isolate DNA from feces for DNA extraction and sequencing. DNA will be subjected to polymerase chain reaction targeting the V3-V4 variable region of bacterial 16S rRNA as a template, followed by sequencing library construction and sequencing reactions on the MiSeq Platform (Illumina, Inc). Sampling will be conducted at day 0, 6 weeks, 12 weeks, and 20 weeks in participants with PD and after screening in healthy participants.

#### Evaluation of Digestive Symptoms

Abdominal examination is a Korean medicine diagnostic method based on the Korean medicine theory that when a disease is inside the body, its response manifests on the body surface [32]. Participants will undergo manual palpation of Zhongwan (CV12) and Tianshu (ST25) for tenderness, while the presence or absence of gastric stuffiness and rigidity below the heart will be checked. The GSRS is a globally used self-reported gastrointestinal symptom scale that covers upper and lower gastrointestinal symptoms [33]. It comprises 15 questions in 5 domains (abdominal pain, reflux syndrome, indigestion syndrome, diarrhea syndrome, and constipation syndrome) scored on a 7-point scale from 1 (no symptoms) to 7 (very severe symptoms). The TDS has been used in previous studies to assess functional dyspepsia [34,35] and comprises 8 items (postprandial fullness and bloating, early satiety, epigastric pain, epigastric burning, nausea, vomiting, belching, and other symptoms) rated on a scale of 0 (absent) to 3 (severe). The BSS is the most widely used measure of stool formation, ranging from the hardest (type 1) to the softest (type 7) [36]. Evaluation will be conducted at day 0, 6 weeks, 12 weeks, and 20 weeks in participants with PD and after screening in healthy participants.

#### Other Evaluations

The MYMOP is a 4-item self-reported health assessment tool that measures subjective symptom change in response to treatment [37]. Using a scale of 0 (as good as it could be) to 6 (as bad as it could be), participants rate the 1 or 2 symptoms that have worried them the most, important activities, and general well-being in the past week. It will be conducted at day 0, 6 weeks, 12 weeks, and 20 weeks in participants with PD and after screening in healthy participants. The credibility assessment in acupuncture trials assesses the patient's credibility of acupuncture treatment, rating 4 items on a scale of 1 (absolutely not) to 6 (absolutely yes) [38]. This tool will be used to evaluate the impact of psychological factors on the outcomes of patients' credibility of acupuncture and moxibustion treatment in this study. This assessment will be conducted at day 0, 6 weeks, and 12 weeks in participants with PD and after screening in healthy participants.

### Safety Assessments and Monitoring

At each visit, the participants’ subjective and objective adverse events will be investigated and classified into mild, moderate, and severe as follows: mild, participant feels little to no discomfort and symptoms interfere little with usual activities of daily living (ADL), most likely not requiring treatment; moderate, participant may experience discomfort that interferes with usual ADL, which may allow the participant to continue the study but may require treatment; severe, participant feels very uncomfortable, interfering with usual ADL, unable to continue participating in the study, and may require treatment or hospitalization. The causal relationship between reported adverse events, acupuncture treatments, and blood collection will be categorized by the Korean medicine doctor in charge as follows: more than 90%, definitely; 70%-90%, probably; 50%-70%, possibly; 30%-50%, probably not; and less than 10%, definitely not.

### Sample Size

This study is not intended to statistically validate the intervention effect of acupuncture and moxibustion on PD but was rather conducted as an exploratory study comparing gut microbiome markers in patients with PD and healthy participants and the effects of acupuncture and moxibustion on the gut microbiome in patients with PD. Therefore, the sample size required for the study will be 60 patients with PD and 20 healthy individuals, depending on the financial resources and study environment, and in consultation with experts in gut microbiology.

### Statistical Analysis

#### Overview of Statistical Analysis

SPSS (ver 21.0, IBM SPSS Statistics for Windows) will be used for analysis. All measures will be presented as mean (SD) for continuous variables and as n (%) for dichotomous variables. For efficacy endpoints, the amount of change or rate of change from baseline to end of treatment and follow-up will be calculated.

All data obtained from participants will be analyzed in 2 forms: intention-to-treat (ITT) analysis and per-protocol (PP) analysis. The ITT analysis will be performed on all participants who undergo acupuncture and moxibustion at least once. For missing data on the evaluation variables, the last observation carried forward (LOCF) method will be applied. PP analysis will further be performed on participants who completed the entire study. The evaluation will be based on the results of the ITT analysis and is presented with reference to the PP analysis.

#### Demographic Information Analysis

For both patients with PD and healthy participants, continuous data will be presented as mean (SD) or as median, minimum, maximum, and 95% CIs, whereas categorical data will be shown as frequencies and percentages.

#### Analysis for Changes in Clinical Outcomes

For the following clinical outcomes with continuous variables, the mean (SD), median, minimum, maximum, and maximum of each outcome will be presented for change in score from day 0 to 6 weeks, 12 weeks, and 20 weeks, and a 1-way repeated measures ANOVA will be performed to test for statistically significant changes in any of the following factors: total MDS-UPDRS score, total MDS-UPDRS score part I score, total MDS-UPDRS part II score, total MDS-UPDRS part III score, total MDS-UPDRS part IV score, BBS, TUG, S&E, PDQ-39, levodopa dosage investigation, abdominal examination, GSRS, TDS, BSS, and MYMOP.

#### Changes in Gut Microbiome–Related Metabolites and Gut Microbiome

Differences in microbiome at each time point will be measured using weighted-unweighted UniFrac distance metrics and subsequently tested using analysis of similarity (ANOSIM). Correlations of clinical outcomes and treatment effects with key microbiomes will be analyzed using Spearman rank correlation analysis, with *P*<.05 considered significant. Principal component analysis will be applied to analyze metabolite distribution in the sample. Partial least squares-discriminant analysis will be applied to identify differences between patients with PD and healthy participants when analyzing metabolite distributions. Selected gut microbiome and metabolite changes will be analyzed by 1-way repeated measures ANOVA, with significant differences between time points determined at *P*<.05. The pathway analysis or enrichment analysis will be performed to analyze the metabolic pathways of the selected metabolites. The Kyoto Encyclopedia of Genes and Genomes database [39] will be used to identify interactions between metabolites. Receiver operating characteristic analysis will be conducted to confirm whether the selected metabolites reflect the representativeness of the disease or treatment.

#### Comparative Analysis of the Gut Microbiome Between Healthy Participants and Patients With PD

Differences in gut microbiome between healthy participants and patients with PD will be determined by testing the acquired data for normality and performing a parametric test with an independent *t* test if the population is normal or a nonparametric test with the Mann-Whitney *U* test if the population is not normal.

### Safety Analysis

For all adverse events that occurred during treatment, the frequency of adverse events will be analyzed according to whether they were associated with treatment or not. The number and proportion of participants with at least one adverse event, 95% CI, and the number of occurrences will be presented.

### Ethical Considerations

The study protocol was approved by the institutional review board of Kyung Hee University Korean Medicine Hospital (KOMCIRB 2021-07-002-001; approved on September 14, 2021). This study is conducted in accordance with the principles of the Declaration of Helsinki. Participants who wish to participate receive an explanation of the consent form from the study researcher and give written consent of their own free will. All data from participants will be simultaneously recorded in an eCRF with an identification code rather than the participant's name to protect personal information.

## Results

The study was supported by the National Research Foundation of Korea, funded by the Ministry of Science and ICT (no. 2022M3A9B6017813). Recruitment for the registry commenced on October 15, 2021, and was scheduled to end on September 13, 2023. Recruitment began on October 22, 2021, and as of February 19, 2025, participant recruitment and observation ended, and data analysis is being conducted.

## Discussion

The clinical efficacy of acupuncture for PD has been consistently reported. Pereira et al [40] analyzed 17 randomized controlled trials (RCTs) on acupuncture for PD published in English between January 2011 and July 2021 and found that the combination of acupuncture and conventional treatment was superior to conventional treatment alone for motor and nonmotor symptoms of PD. Similarly, Lee et al [15] analyzed 16 RCTs, investigating 15 acupuncture treatments and 1 moxibustion treatment, published between 2008 and 2013, and comparing acupuncture or moxibustion with conventional therapies such as dopamine, found that all but one showed significant improvement in the acupuncture or moxibustion treatment group. In particular, for nonmotor symptoms of PD, for which effective treatments remain lacking, Li et al [41] reported through an analysis of 27 RCTs that acupuncture treatment could improve depression, quality of life, cognition, total mentation, behavior and mood, and ADL in patients with PD. Based on these clinical study data, the Clinical Practice Guideline of Korean Medicine for Parkinson's Disease recommended combination treatment of acupuncture and anti-Parkinsonian drugs for patients with PD, while moxibustion treatment may also be considered [42].

Attempts to identify the mechanisms of action of acupuncture in PD to support clinical data are still ongoing. Previous experimental studies using PD models have shown that acupuncture exerts neuroprotective effects through activation of neuroprotective agents such as brain-derived neurotrophic factor, glial cell line-derived neurotrophic factor, and cyclophilin A [43], effective in PD symptoms by inducing neuromodulation, thus controlling inflammation and brain functional connectivity [44,45], and p53 signaling-mediated neuroprotective effect [46]. However, these opinions are insufficient to reflect the systemic nature of PD and do not fully explain the improvement in nonmotor symptoms associated with acupuncture.

The gut-brain axis mechanism of PD stemmed from the perspective that PD should be managed and treated as a systemic disease [7]. Braak et al [47] observed that unknown pathogens invade the body through the intestines, while Lewy bodies selectively move from the intestines along the central nervous system and metastasize to the brain; based on the hypothesis that the motor and nonmotor symptoms of PD appear according to this metastasis, they proposed the Braak stage, which divides this process into 6 stages. This finding has drawn attention to the role of the gut in the pathology of PD. Recently, it has also been hypothesized that PD is divided into brain-first and gut-first, based on the division of positive and negative rapid eye movement sleep behavior disorder in the prodromal phase [48]. The fact that gastrointestinal symptoms, such as constipation, appear very early in the prodromal phase [49] and that 60%-80% of patients experience gastrointestinal symptoms [50] may also support the gut-first PD hypothesis.

Imbalances in the gut-brain axis caused by gut microbial dysbiosis can lead to increased inflammatory signaling and epithelial permeability, both of which are known to play an important role in neurodegenerative diseases such as PD [19]. The neurotoxin 1-methyl-4-phenyl-1,2,3,6-tetrahydropyridine increases α-synuclein and influences compositional changes in gut microbiome, including the phylum Proteobacteria [51]. Gut microbiome dysbiosis also induces microglial activation and neuroinflammation, which may promote α-synuclein overexpression, resulting in motor dysfunction [52,53]. Taken together, these results suggest that regulation of gut microbiome may have significant potential as a key treatment for PD, functioning through modulation of the gut-brain axis.

In the TEAM theory, the functional connection and communication between acupoints, the brain (ancient heart), and the gut is clinically important [54]. Based on the traditional theory, a previous review study [55] has reported that acupuncture has an effect on various diseases, including metabolic diseases, gastrointestinal diseases, mental disorders, and nervous system diseases, by regulating gut microbiome. Further, a large number of experimental studies on the relationship between PD and gut microbiome have been published recently, and the number is rapidly increasing [55]. In a previous experimental study including a PD model [19], we found that acupuncture treatment with GB34, which has been shown to exert neuroprotective effects on the brain, and ST36, which has been shown to enhance digestive function, improved motor function and anxiety, and increased the levels of dopaminergic fibers and neurons in the striatum and substantia nigra, increased the diversity of gut microbiome (alpha-diversity) and restored a gut microbiome to a composition more similar to that of robust controls (beta-diversity) compared with that noted in the PD group, in addition to restoring the increased levels of proteobacteria at the phylum level, suggesting that acupuncture exerts effects on improving gut microbiome imbalances. In another previous study [56], we conducted a clinical study to investigate whether acupuncture treatment for 2 weeks could effectively improve motor symptoms of PD using the UPDRS II+III in 73 patients with PD. This study showed that both the acupuncture treatment group and the control group showed improvement in motor function, with no significant intergroup difference; however, at the follow-up of 2 months after the end of treatment, the acupuncture treatment group continued to maintain these improvements in symptoms, while the control group reported a regression. Considering the results of these previous experimental studies, we hypothesized that acupuncture in this clinical study affected changes in gut microbiome to improve PD symptoms in a sustainable manner. To verify this hypothesis, we present the protocol of this study to explore changes in gut microbiome caused by acupuncture and moxibustion in patients with PD and explore its association with improvement in clinical symptoms of PD.

This protocol has limitations. First, as this study was designed as a single-group, prospective observational study with a primary focus on analyzing pre-post changes of patients with PD, a control group of healthy participants was included for baseline comparison rather than incorporating a control group within the PD cohort. This limits the internal validity of the intervention findings, as changes may be influenced by natural disease progression or placebo effects rather than the treatment itself. Second, although the sample size was determined based on feasibility rather than statistical power calculations, 60 patients with PD and 20 healthy participants may limit the reliability and generalizability of both within-group and between-group comparisons and increase the risk of type II error. Third, as no blinding was applied, the use of subjective, patient- and clinician-reported outcomes such as MDS-UPDRS, PDQ-39, and MYMOP increases the risk of detection and expectation bias, potentially compromising causal inference in this single-arm design. Fourth, adverse events are assessed by the treating Korean medicine doctors without independent adjudication or standardized grading criteria, which may limit the objectivity and consistency of safety reporting in this study. To address these limitations, future studies should adopt an RCT design with an adequately powered sample size, blinding procedures, and standardized outcome and safety assessments to enhance the internal validity, causal inference, and generalizability.

This study has limitations that must be weighed when interpreting the findings. Nutritional management is recommended for patients with PD, including dietary distribution to optimize levodopa absorption, dietary interventions for the improvement of nonmotor symptoms, and regular monitoring of food intake [57]. This leads to differences in dietary habits between PD patients and robust people, with PD patients showing higher protein and higher fiber dietary habits than robust people [58]. Fibers activate various beneficial microbiomes and suppress harmful species, and protein also has a significant impact on the gut microbiome [59]. Since this study does not investigate the nutritional intake of PD patients and healthy individuals, the influence of dietary differences should be considered when comparing the gut microbiome results between the two groups. Furthermore, levodopa dosage may affect gut microbiome composition [60]. Although this study investigates levodopa dosage in PD patients as part of clinical outcomes, it does not exclude the potential confounding effect of dosage adjustment on the gut microbiome. Therefore, any significant changes in levodopa dosage should be taken into consideration in the results.

The study will generate data on the effects of acupuncture and moxibustion on gut microbiome of patients with PD and explore its association with improvement in clinical symptoms. If the study results demonstrate that acupuncture and moxibustion change the gut microbiome in patients with PD, it will be the first clinical evidence to support a microbiome-gut-brain axis mechanism for acupuncture and moxibustion treatment of PD, ultimately contributing to the potential of acupuncture and moxibustion as an alternative treatment for PD.

## Appendix

Multimedia Appendix 1 Planned schedule for the enrollment, interventions, and assessments of patients with Parkinson disease.

Multimedia Appendix 2 SPIRIT (Standard Protocol Items: Recommendations for Interventional Trials) checklist.

## Abbreviations

ADL: activities of daily living
ANOSIM: analysis of similarity
BBS: Berg Balance Scale
BSS: Bristol Stool Scale
CAM: complementary and alternative medicine
CRF: case report form
GSRS: Gastrointestinal Symptom Rating Scale
HrQoL: health-related quality of life
ITT: intention to treat
LOCF: last observation carried forward
MDS-UPDRS: Movement Disorder Society–Sponsored Revision of the Unified Parkinson's Disease Rating Scale
MYMOP: Measure Yourself Medical Outcome Profile
PD: Parkinson disease
PDQ-39: 39-Item Parkinson's Disease Questionnaire
PP: per-protocol
RCT: randomized controlled trial
S&E: Schwab and England Activities of Daily Living Scale
SPIRIT: Standard Protocol Items: Recommendations for Interventional Trials
STRICTA: Standards for Reporting Interventions in Clinical Trials of Acupuncture
TDS: Total Dyspepsia Symptom Scale
TEAM: Traditional East Asian Medicine
TUG: Timed Up and Go Test
UPDRS: Unified Parkinson's Disease Rating Scale

## Data Availability

Data sharing does not apply to this protocol, as no datasets have been collected or analyzed.
