# Supplementary material for: Changes in Gut Microbiome Following Acupuncture and Moxibustion in Patients With Parkinson Disease: Protocol for a Single-Group, Prospective, Observational Study
Source: JMIR Res Protoc. 2025 Oct 29;14:e76551. doi: 10.2196/76551 (PMC12612644; doi:10.2196/76551)
Supplement: Multimedia Appendix 1 [file resprot_v14i1e76551_app1.docx]

**Table S1.** Planned schedule for the enrollment, interventions, and assessments of patients with Parkinson disease.

| Visit | 0 | 1 | 2 | 3 | 4 | 5 | 6 | 7 | 8 | 9 | 10 | 11 | 12 | 13 | 14 | 15 | 16 | 17 | 18 | 19 | 20 | 21 | 22 | 23 | 24 | 25 | UV^a^ |
| --- | --- | --- | --- | --- | --- | --- | --- | --- | --- | --- | --- | --- | --- | --- | --- | --- | --- | --- | --- | --- | --- | --- | --- | --- | --- | --- | --- |
| Day | –14 to 0 | 0 | ~7 | ~14 | ~14 | ~21 | ~21 | ~28 | ~28 | ~35 | ~35 | ~42 | ~42 | ~49 | ~49 | ~56 | ~56 | ~63 | ~63 | ~70 | ~70 | ~77 | ~77 | ~84 | ~84 | — | — |
| Week | 0 | 1 | 1 | 2 | 2 | 3 | 3 | 4 | 4 | 5 | 5 | 6 | 6 | 7 | 7 | 8 | 8 | 9 | 9 | 10 | 10 | 11 | 11 | 12 | 12 | 20 | — |
| Visit window | — | — | ±2 | ±2 | ±2 | ±2 | ±2 | ±2 | ±2 | ±2 | ±2 | ±2 | ±2 | ±2 | ±2 | ±2 | ±2 | ±2 | ±2 | ±2 | ±2 | ±2 | ±2 | ±2 | ±2 | ±7 | — |
|  |  |  |  |  |  |  |  |  |  |  |  |  |  |  |  |  |  |  |  |  |  |  |  |  |  |  |  |
| Informed content | ✓ |  |  |  |  |  |  |  |  |  |  |  |  |  |  |  |  |  |  |  |  |  |  |  |  |  |  |
| Demographic information | ✓ |  |  |  |  |  |  |  |  |  |  |  |  |  |  |  |  |  |  |  |  |  |  |  |  |  |  |
| Medical history | ✓ |  |  |  |  |  |  |  |  |  |  |  |  |  |  |  |  |  |  |  |  |  |  |  |  |  |  |
| Parkinson disease diagnosis and treatment history | ✓ |  |  |  |  |  |  |  |  |  |  |  |  |  |  |  |  |  |  |  |  |  |  |  | ✓ | ✓ |  |
| Medications investigation | ✓ |  |  |  |  |  |  |  |  |  |  |  |  |  |  |  |  |  |  |  |  |  |  |  | ✓ | ✓ |  |
| Vital sign | ✓ | ✓ | ✓ | ✓ | ✓ | ✓ | ✓ | ✓ | ✓ | ✓ | ✓ | ✓ | ✓ | ✓ | ✓ | ✓ | ✓ | ✓ | ✓ | ✓ | ✓ | ✓ | ✓ | ✓ | ✓ | ✓ | ✓ |
| Hoehn and Yahr stage | ✓ |  |  |  |  |  |  |  |  |  |  |  |  |  |  |  |  |  |  |  |  |  |  |  |  |  |  |
| Evaluating inclusion and exclusion criteria | ✓ |  |  |  |  |  |  |  |  |  |  |  |  |  |  |  |  |  |  |  |  |  |  |  |  |  |  |
| MDS-UPDRS^b^ |  | ✓ |  |  |  |  |  |  |  |  |  |  | ✓ |  |  |  |  |  |  |  |  |  |  |  | ✓ | ✓ | ✓ |
| BBS^c^ |  | ✓ |  |  |  |  |  |  |  |  |  |  | ✓ |  |  |  |  |  |  |  |  |  |  |  | ✓ | ✓ | ✓ |
| TUG^d^ |  | ✓ |  |  |  |  |  |  |  |  |  |  | ✓ |  |  |  |  |  |  |  |  |  |  |  | ✓ | ✓ | ✓ |
| S&E^e^ |  | ✓ |  |  |  |  |  |  |  |  |  |  | ✓ |  |  |  |  |  |  |  |  |  |  |  | ✓ | ✓ | ✓ |
| PDQ-39^f^ |  | ✓ |  |  |  |  |  |  |  |  |  |  | ✓ |  |  |  |  |  |  |  |  |  |  |  | ✓ | ✓ | ✓ |
| Levodopa dosage investigation |  | ✓ |  |  |  |  |  |  |  |  |  |  | ✓ |  |  |  |  |  |  |  |  |  |  |  | ✓ | ✓ | ✓ |
| Blood sampling^g^ |  | ✓ |  |  |  |  |  |  |  |  |  |  | ✓ |  |  |  |  |  |  |  |  |  |  |  | ✓ | ✓ |  |
| Fecal sampling^h^ |  | ✓ |  |  |  |  |  |  |  |  |  |  | ✓ |  |  |  |  |  |  |  |  |  |  |  | ✓ | ✓ |  |
| Abdominal examination |  | ✓ |  |  |  |  |  |  |  |  |  |  | ✓ |  |  |  |  |  |  |  |  |  |  |  | ✓ | ✓ | ✓ |
| GSRS^i^ |  | ✓ |  |  |  |  |  |  |  |  |  |  | ✓ |  |  |  |  |  |  |  |  |  |  |  | ✓ | ✓ | ✓ |
| TDS^j^ |  | ✓ |  |  |  |  |  |  |  |  |  |  | ✓ |  |  |  |  |  |  |  |  |  |  |  | ✓ | ✓ | ✓ |
| BSS^k^ |  | ✓ |  |  |  |  |  |  |  |  |  |  | ✓ |  |  |  |  |  |  |  |  |  |  |  | ✓ | ✓ | ✓ |
| MYMOP^l^ |  | ✓ |  |  |  |  |  |  |  |  |  |  | ✓ |  |  |  |  |  |  |  |  |  |  |  | ✓ | ✓ | ✓ |
| Credibility assessment in trials of acupuncture |  | ✓ |  |  |  |  |  |  |  |  |  |  | ✓ |  |  |  |  |  |  |  |  |  |  |  | ✓ |  |  |
| Acupuncture and moxibustion treatment |  | ✓ | ✓ | ✓ | ✓ | ✓ | ✓ | ✓ | ✓ | ✓ | ✓ | ✓ | ✓ | ✓ | ✓ | ✓ | ✓ | ✓ | ✓ | ✓ | ✓ | ✓ | ✓ | ✓ | ✓ |  |  |
| KM^m^ doctor interview |  | ✓ | ✓ | ✓ | ✓ | ✓ | ✓ | ✓ | ✓ | ✓ | ✓ | ✓ | ✓ | ✓ | ✓ | ✓ | ✓ | ✓ | ✓ | ✓ | ✓ | ✓ | ✓ | ✓ | ✓ | ✓ | ✓ |
| Adverse events investigation |  | ✓ | ✓ | ✓ | ✓ | ✓ | ✓ | ✓ | ✓ | ✓ | ✓ | ✓ | ✓ | ✓ | ✓ | ✓ | ✓ | ✓ | ✓ | ✓ | ✓ | ✓ | ✓ | ✓ | ✓ | ✓ | ✓ |

^a^UV: unscheduled visits. Visits on a day other than the original visit schedule because of an adverse event.

^b^MDS-UPDRS: Movement Disorder Society–Sponsored Revision of the Unified Parkinson's Disease Rating Scale.

^c^BBS: Berg Balance Scale.

^d^TUG: Timed Up and Go Test.

^e^S&E: Schwab and England Activities of Daily Living Scale.

^f^PDQ-39: 39-Item Parkinson's Disease Questionnaire.

^g^Analyzing gut microbiome–related metabolites.

^h^Analyzing gut microbiome.

^i^GSRS: Gastrointestinal Symptom Rating Scale.

^j^TDS: Total Dyspepsia Symptom Scale.

^k^BSS: Bristol Stool Scale.

^l^MYMOP: Measure Yourself Medical Outcome Profile.

^m^KM: Korean medicine.
